# Birds of Antioquia: Georeferenced database of specimens from the Colección de Ciencias Naturales del Museo Universitario de la Universidad de Antioquia (MUA)

**DOI:** 10.3897/zookeys.410.7109

**Published:** 2014-05-21

**Authors:** Andrea Morales Rozo, Fernando Valencia, Alexis Acosta, Juan Luis Parra

**Affiliations:** 1 Instituto de Biología, Grupo de Ecología y Evolución de Vertebrados, Universidad de Antioquia, calle 67 No 53-108, Medellín, Colombia; 2 Museo Universitario de la Universidad de Antioquia, calle 67 No 53-108, Medellín, Colombia

**Keywords:** Antioquia, Aves, Birds, Colombia, Georeference, Museum, Specimens, Universidad de Antioquia

## Abstract

The department of Antioquia, Colombia, lies in the northwestern corner of South America and provides a biogeographical link among divergent faunas, including Caribbean, Andean, Pacific and Amazonian. Information about the distribution of biodiversity in this area is of relevance for academic, practical and social purposes. This data paper describes the dataset containing all bird specimens deposited in the Colección de Ciencias Naturales del Museo Universitario de la Universidad de Antioquia (MUA). We curated all the information associated with the bird specimens, including the georeferences and taxonomy, and published the database through the Global Biodiversity Information Facility network. During this process we checked the species identification and existing georeferences and completed the information when possible. The collection holds 663 bird specimens collected between 1940 and 2011. Even though most specimens are from Antioquia (70%), the collection includes material from several other departments and one specimen from the United States. The collection holds specimens from three endemic and endangered species (*Coeligena orina*, *Diglossa gloriossisima*, and *Hypopirrhus pyrohipogaster*), and includes localities poorly represented in other collections. The information contained in the collection has been used for biodiversity modeling, conservation planning and management, and we expect to further facilitate these activities by making it publicly available.

## Introduction

**Figure 1.**
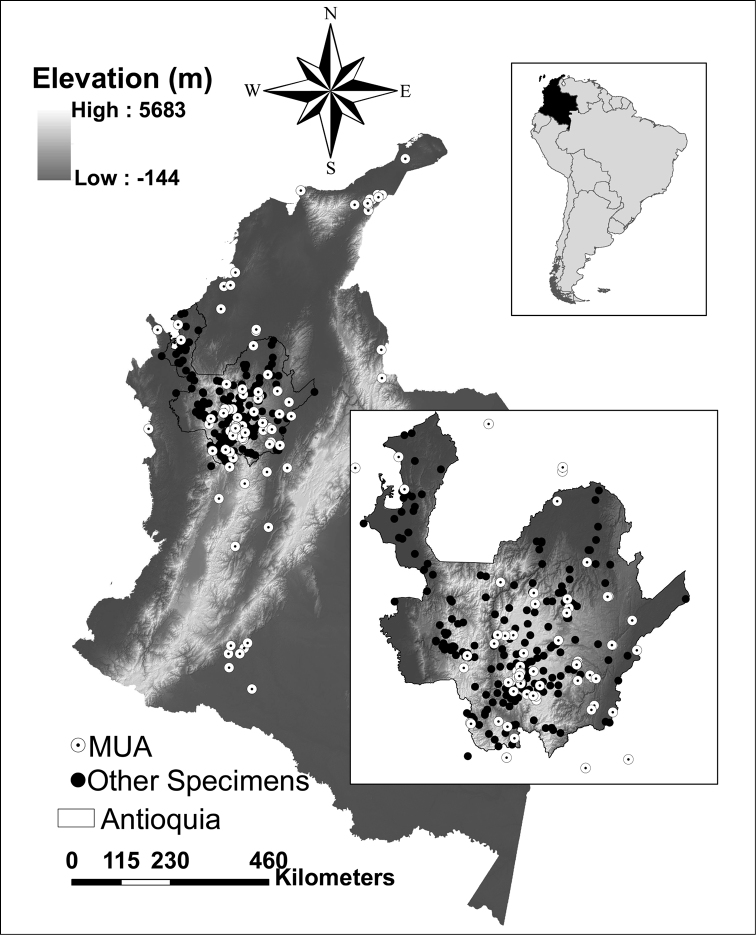
Map of Colombia showing the collection localities for all bird specimens held in the MUA (white dots) and all specimens from Antioquia held in other collections (black dots). The upper right inset highlights Colombia within South America and the lower right inset provides a closer look at the distribution of points in Antioquia.

The department of Antioquia lies in the northwestern corner of Colombia and is one of the most biodiverse regions in the country. Several factors contribute to the concentration of biodiversity within this area, including high environmental heterogeneity and the confluence of the Panamanian and Neotropic zoogeographical regions (Holt et al. 2013). Currently, Antioquia is under severe pressure from mining and hydroelectric projects (Finer and Jenkins 2012). Knowledge of the spatial distribution of biodiversity in the department is of critical importance for assessing the impacts of these activities and deciding where to grant exploitation licenses. Nonetheless, spatially explicit information about the occurrence of birds in this region is relatively unusual, apart from data from museum collections. However, most of this information is still not available in the public domain in databases such as ORNIS or global networks such as GBIF (Fig. 1). Thus, our primary motivation is to make the information present in the collection of the Universidad de Antioquia available for the general public. Despite its small size (663 specimens), the collection holds specimens that are rare in other collections, including three endemic and endangered birds, and localities that have been poorly sampled such as the eastern flank of the Central Andes (Fig. 1). By making this information available we expect it to be further used in academic endeavors such as ecological niche modeling (e.g., Velásquez-Tibatá et al. 2012) and for practical purposes related to conservation planning and management (e.g., Krabbe et al. 2005).

## Project details

**Project title:** Geographic distribution of species richness in the Colombian Andes with a special emphasis on aquatic systems of the northwestern region.

**Personnel:** Andrea Morales Rozo (research assistant, data collector), Juan Luis Parra (principal investigator), Fernando Valencia (collection manager), Alexis Acosta (collection coordinator, data collector).

**Funding:** The Nature Conservancy (TNC).

## Taxonomic coverage

**General taxonomic coverage description:** The collection holds specimens from 313 species, 54 families and 19 orders (Fig. 3). Nonetheless, this is still a poor representation of the total number of species in Colombia (~17%) and the department of Antioquia (~37%). The three most represented families are Thraupidae (41 species, 96 specimens), Tyrannidae (35, 63) and Trochilidae (33, 69). Specimens of particular relevance in this collection are from Terenura callinota and Spizastur melanoleucus, representing the only specimens of these species in Antioquia. In addition, the collection includes specimens from three endemic and endangered species: Coeligena orina (1 specimen, CR), Diglossa gloriossisima (1, EN) and Hypopirrhus pyrohipogaster (2, VU).

## Taxonomic ranks

**Kingdom:** Animalia

**Class:** Aves

**Order:** Accipitriformes, Anseriformes, Apodiformes, Caprimulgiformes, Charadriiformes, Columbiformes, Coraciformes, Cuculiformes, Falconiformes, Galbuliformes, Galliformes, Gruiformes, Passeriformes, Pelecaniformes, Piciformes, Podicipediformes, Psittaciformes, Strigiformes, Suliformes.

**Family:** Accipitridae, Alcedinidae, Anatidae, Anhingidae, Aramidae, Ardeidae, Bucconidae, Capitonidae, Caprimulgidae, Cardinalidae, Charadriidae, Columbidae, Conopophagidae, Corvidae, Cotingidae, Cracidae, Cuculidae, Donacobiidae, Emberizidae, Falconidae, Formicariidae, Fringillidae, Furnariidae, Galbulidae, Grallaridae, Hirundinidae. Icteridae, Incertae sedis, Jacanidae, Mimidae, Momotidae, Odontophoridae, Parulidae, Phalacrocoracidae, Picidae, Pipridae, Podicipedidae, Psittacidae, Rallidae, Ramphastidae, Recurvirostridae, Rhinocryptidae, Steatornithidae, Strigidae, Thamnophilidae, Thraupidae, Threskiornithidae, Tityridae, Trochilidae, Troglodytidae, Turdidae, Tyrannidae, Tytonidae, Vireonidae.

**Common names:** Birds.

## Spatial coverage

**General spatial coverage:** The sampling localities range from 71°S to 78°S longitude and from 0°N to 12°N latitude. All specimens are from Colombia except one from the United States. The dataset represents an improvement over the area sampled within Antioquia, in particular the eastern part of the department where no specimens exist in other collections (Fig. 1). Clearly, there are gaps in collection effort throughout the department, but we highlight the Pacific lowlands, the Western Andes and the lowlands in the northern part of the Central and Western Andes as areas that should be the focus of biodiversity expeditions.

**Coordinates:** 0°0'0"N and 12°0'0"N Latitude; 71°0'0"W and 78°0'0"W Longitude.

**Temporal coverage:** Specimens in the collection date from 1940 to 2011 with two clear increments during the early 1970s and 2000s (Fig. 3). The most recent peak in collections traces to the establishment of a large project focused on cataloguing the department´s biodiversity, and the early peak is related to a successful collaboration between the curators of the largest collections in the department of Antioquia (MUA and ITM).

## Natural collections description

**Parent collection identifier:** Museo Universitario de la Universidad de Antioquia

**Collection name:** Colección de Aves

**Collection identifier:** Fernando Valencia

**Specimen preservation method:** Skin

## Methods

**Figure 2.**
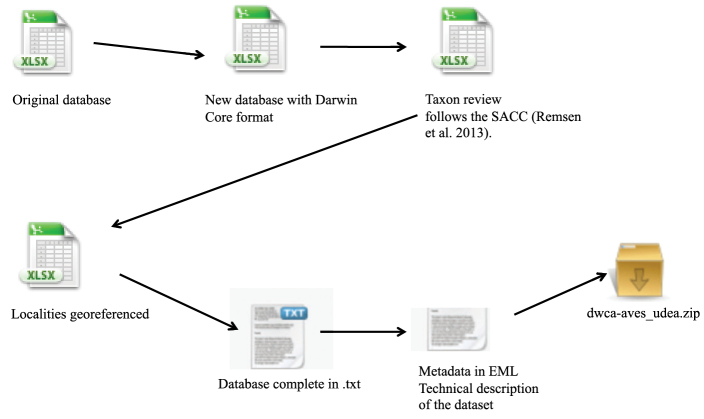
Schematic flowchart of the steps taken to format the collection database according to the Darwin Core and submitting it for public access through GBIF.

**Figure 3.**
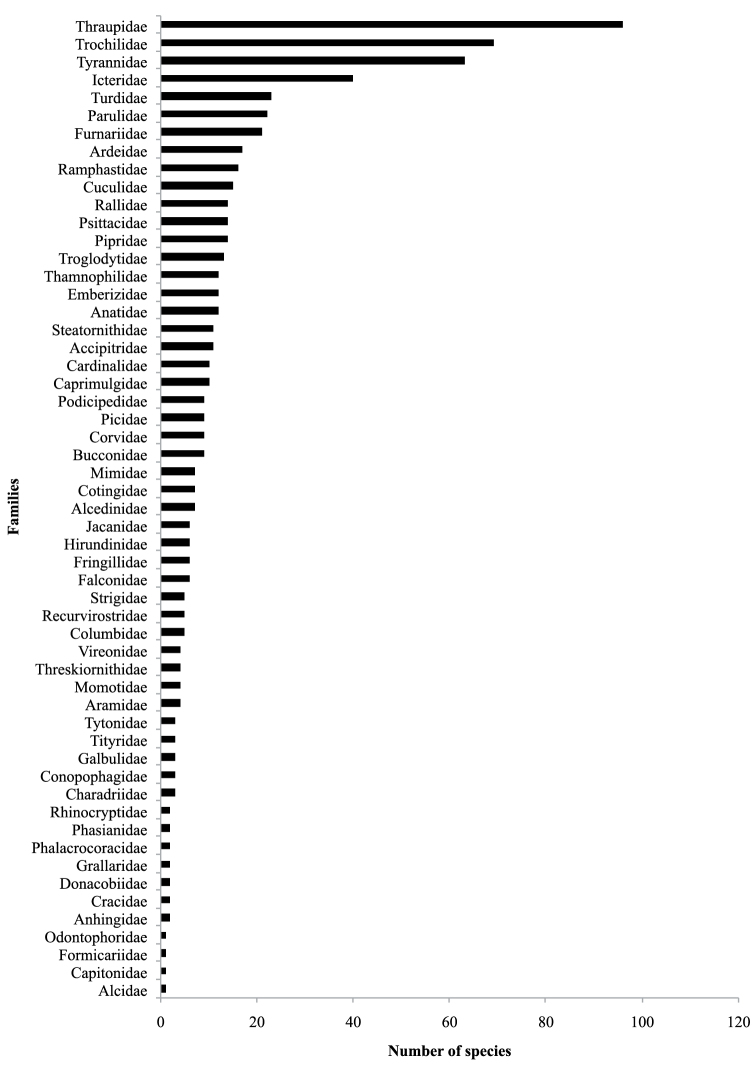
Distribution of specimens in the collection according to families.

**Method step description:** The database of bird specimens was developed with the aim of determining the current distribution of avian richness along major river banks. Birds are a particularly useful taxon for conservation assessments since they are easy to identify and are one of the best-known groups in terms of their distribution and abundance (Blair 1999). Many bird species concentrate along river banks and other water bodies and can be used as indicators of ecosystem health (Péron et al. 2013). To obtain a georeferenced database for all specimens in the collection, we followed the procedure represented in Figure 3. We initiated by organizing the information present in the original collection database according to the biodiversity information standards established in Darwin Core 2.0. Each specimen was identified by AMR and all localities specified in the specimen labels were georeferenced using gazeteers (Paynter 1997), maps from the National Institute of Geography (IGAC), Google Earth (URL: http://www.google.com/earth/) and Geonames (URL: http://www.geonames.org/). Finally, we published the database to the GBIF network through the Humboldt Institute, the Colombian GBIF node (Fig. 2). After we finished data collection and validation, we mapped the specimen localities and compared them to other museums (Fig. 1).

**Figure 4.**
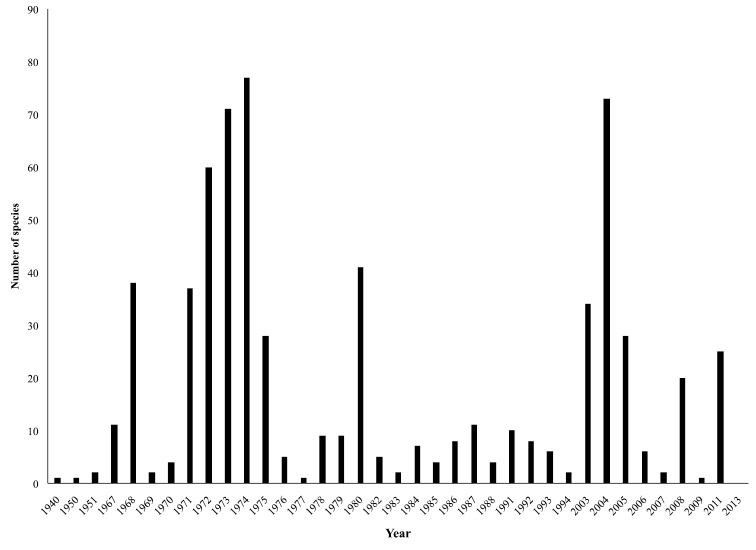
Distribution of specimens through time, showing two peaks in collection activity during the early 1970s and 2000s. Both time periods correspond to the timing of large-scale projects.

**Study extent description:** The department of Antioquia lies in the northwestern corner of Colombia close to the border with Panama. Most of the bird specimens in the collection (459) are from within the department, with the following exceptions: Córdoba (69 specimens), Caquetá (46), Amazonas (3), Caldas (6), Chocó (12), La Guajira (28), Magdalena (9), Meta (6), Norte de Santander (2), Sucre (2), Tolima (4), and Valle del Cauca (7). One specimen is from the United States and nine specimens only listed Colombia in the locality information (Fig. 1). The area encompassed by Antioquia is of high relevance to biodiversity conservation due to its biogeographic position (Haffer 1967), its high environmental heterogeneity, and its current threat due to mining and hydroelectric pressures (Holt et al. 2013). This area contains a high number of Colombian endemics and a concentration of range restricted and endangered birds (Velasquez-Tibatá et al. 2012). The department of Antioquia encompasses a wide range of environments, including dry forests in the Magdalena and Cauca river valleys, wet lowland forests in the Pacific and montane forests and páramo on the Central and Western Andes (Toro-Murillo and Cuervo-Maya 2002). Despite its great significance as a biodiversity hotspot in Colombia, Antioquia is also recognized for its high rates of deforestation (Orrego 2009).

**Sampling description:** The specimens held in the collection come from a variety of sources including field expeditions from the curators, research projects focused on particular species and private donations. Thus, there is no single collection or specimen preparation protocol: Nonetheless, most specimens have been captured through the use of mist nets and the majority of specimens are complete skins.

**Quality control description:** An experienced ornithologist (AMR) identified all specimens with the aid of field guides (Del-Hoyo et al. 1999, Hilty and Brown 1986, McMullan et al. 2010). The taxonomy of each bird was updated to reflect the South American Classification Committee version 20 May 2013 (Remsen et al. 2013). Each georeference was considered in light of the species distribution given by Birdlife International (Ridgely et al. 2012) and elevation ranges according to Hilty and Brown (1986) and McMullan et al. (2010). We used this information in addition to the georeferencing protocols suggested by Chapman and Wieczcoreck (1999) to estimate the uncertainty of the georeference whenever possible. There were 46 specimens that could not be georeferenced given the information on the label.

## Dataset description

**Object name:** Darwin Core Archive for the Database of Birds of MUA

**Character encoding:** UTF-8

**Format name:** Darwin Core Archive format

**Format version:** 1.0

**Distribution:** http://ipt.sibcolombia.net/sib/resource.do?r=aves_udea

**Publication date of data:** March 31st 2014

**Language:** Spanish

**Metadata language:** Spanish

**Date of metadata creation:** March 31st 2014

**Hierarchy level:** Dataset
